# Clinical and surgical profiles of patients submitted to plastic surgery procedures after bariatric surgery in a public hospital in Brazil’s Midwest

**DOI:** 10.1590/0100-6991e-20253812-en

**Published:** 2025-07-31

**Authors:** JEFFERSON LESSA SOARES DE MACEDO, SIMONE CORRÊA ROSA, LUIS FELIPE ROSA DE MACEDO, CECÍLIA ROSA DE MACEDO, MARIANA FIUZA GONÇALVES, BRENNER DOLIS MARRETTO DE MOURA

**Affiliations:** 1 - Hospital Regional da Asa Norte,, Unidade de Cirurgia Plástica - Brasília - DF - Brasil; 2 - FEPECS, Escola Superior de Ciências Da Saúde - Brasília - DF - Brasil; 3 - Universidade Católica de Brasília, Curso de Graduação em Medicina - Brasília - DF - Brasil; 4 - UNICEUB, Curso de Graduação em Medicina - Brasília - DF - Brasil; 5 - Hospital Regional da Asa Norte, Unidade de Cirurgia Geral - Brasília - DF - Brasil

**Keywords:** Surgery, Plastic, Plastic Surgery Procedures, Abdominoplasty, Postoperative Complications, Bariatric Surgery, Cirurgia Plástica, Cirurgia Bariátrica, Complicações Pós-Operatórias, Abdominoplastia, Procedimentos de Cirurgia Plástica

## Abstract

**Introduction:**

Patients who undergo to gastroplasty present massive weight loss and the plastic surgery represents an important play in the treatment. The aim of this study is to present the profile of patients who underwent plastic surgery after bariatric surgery performed at the Reference Public Hospital in West-Center of Brazil.

**Methods:**

A descriptive, analytical and retrospective study was performed in a single public hospital on patients who underwent post-bariatric plastic surgery from January 2011 to December 2023. Three hundred and sixteen patients who underwent plastic surgery following Roux-Y gastroplasty were studied. Measures included BMI (body mass index) before gastroplasty and before plastic surgery, medical complications and comorbidities.

**Results:**

316 patients (297 female, 19 male) with a mean age of 43 years underwent 268 separated operations. The average BMI at the time of plastic surgery was 27,39kg/m^2^ . Average weight loss was 47,44kg and mean pre-weight loss BMI (max BMI) was 45,5kg/m^2^ .The most important preplastic comorbities were: arterial hypertension (12,7%), degenerative artrophaty (7,0%), diabete melito (5,7%) and methabolic syndrom (4,4%). From 316 patients operated, 75,7% were underwent abdominoplasty followed by mammaplasy (41,4%), ritidoplasty (12,0%), and brachioplasty (12,0%). Thirty-nine (12,3%) patients had hernia repair in combination with abdominoplasty. The complication rate was 31,3%.

**Conclusion:**

Epidemiological profile of postbariatric patients who underwent body contour surgery showed peculiar clinical, anthropometric and surgical aspects, specially the low prevelence of comorbidities, the low number of associated surgeries and rate of postoperative complications in the group studied.

## RESUMO

**Introdução:**

Pacientes submetidos à gastroplastia evoluem com grande perda de peso e a cirurgia plástica tem papel importante no tratamento do excesso de pele. O objetivo deste estudo é avaliar o perfil clínico e cirúrgico dos pacientes pós-bariátricos operados em um hospital público de referência do centro-oeste do Brasil.

**Métodos:**

Trata-se de estudo descritivo, analítico e retrospectivo realizado em um único hospital público com pacientes pós-bariátricos submetidos a procedimentos em cirurgia plástica após gastroplastia em Y-Roux no período de janeiro de 2011 a dezembro de 2023. As variáveis analisadas incluíram: IMC (Índice de Massa Corporal) antes e depois da gastroplastia, presença de comorbidades e taxa de complicações.

**Resultados:**

A amostra estudada foi de trezentos e dezesseis pacientes. Sendo 297 (94%) mulheres com média de idade de 43 anos submetidas a 268 operações únicas. A média do IMC no momento da cirurgia plástica foi 27,39kg/m^2^. A média da perda de peso foi 47,44kg e IMC máximo foi 45,5kg/m^2^. Principais comorbidades pré-plástica foram: hipertensão arterial (12,7%), artropatia (7,0%), diabete melito (5,7%) e síndrome metabólica (4,4%). Os principais procedimentos nos pacientes pós-bariátricos foram: abdominoplastia (75,7%), mastoplastia (41,4%), ritidoplastia (12,0%) e braquioplastia (12,0%). Trinta e nove (12,3%) pacientes foram submetidos à herniorrafia associado abdominoplastia. A taxa geral de complicações pós-operatórias foi 31,3%.

**Conclusão:**

Perfil epidemiológico dos pacientes pós-bariátricos que se submeteram a procedimentos em cirurgia plástica apresentaram características clínicas, antropométrica e cirúrgica específicas, especialmente na baixa prevalência de comorbidades residuais, no número de cirurgias associadas e taxa de complicações pós-operatórias no grupo estudado.

## INTRODUCTION

According to the World Health Organization, there are more than two billion overweight people in the world and obesity is present in 650 million individuals^1^. In Brazil, the data are not far from expectations: one in four Brazilians have obesity^2^, demonstrating that its high prevalence has become a global public health problem, demanding great concern from the health sectors^3^.

Obesity is associated with several important comorbidities, such as diabetes, hypertension, dyslipidemia, obstructive sleep apnea, and cardiovascular diseases, and can lead to a reduction in life expectancy of up to 20 years^4^. Among the treatments for this condition, success for a limited time has been observed with behavioral and pharmacological methods, while bariatric surgery has been the most popular and successful in recent times, as it is effective in substantial and sustained weight loss, and correlated with the improvement of related comorbidities^4^.

A common complaint among patients undergoing surgery for the treatment of obesity is dysmorphism due to excess skin and adipose tissue remaining from weight loss, leading to emotional fragility with low self-esteem, mood swings, skin rubbing infections, leading them to seek subsequent plastic surgery procedures for remodeling and removal of excess skin^5^.

Knowledge of the epidemiological, clinical, and surgical profile of post-bariatric patients undergoing plastic surgery procedures is essential for the planning of public policies aimed at the treatment of these patients. In addition, it provides adequate preoperative preparation and potential reduction of postoperative complications. The study aims to analyze the epidemiological, clinical, and surgical profile of post-bariatric patients who underwent reconstructive procedures in plastic surgery.

## METHODS

This is a retrospective cohort study, conducted in a public referral hospital for bariatric and plastic surgery in the Brazilian Midwest. We evaluated individuals who had been submitted to Roux-en-Y gastric bypass and who subsequently underwent plastic surgery procedures between January 2011 and December 2023, after massive weight loss. The indications for bariatric surgery followed the recommendations for obesity surgery of the International Federation for the Surgery of Obesity and Metabolic Diseases^6^.

The initial sample consisted of 341 patients who sought reconstructive procedures after massive weight loss in the Plastic Surgery Unit of a public hospital in the Midwest of Brazil. We excluded 25 patients from the study based on the exclusion criteria (nine patients due to smoking, five due to gestational intention, five due to incomplete data, and six patients due to weight instability). The final sample consisted of 316 post-bariatric patients who underwent plastic surgery procedures after Roux-en-Y gastroplasty. The inclusion and exclusion criteria are described in Table 1.

**Table 1:** Study inclusion and exclusion criteria.

Inclusion criteria	Exclusion criteria
Weight stability for at least six months after the weight loss goal has been achieved	Smoking, gestational intention, weight instability, with non-maintenance of weight for six months
Absence of illicit drug use or alcoholism	Individuals who have not signed the ICF
Absence of moderate or severe psychosis or dementia	Patients undergoing other bariatric procedures after Roux-en-Y gastroplasty
Postoperative follow-up of at least six months	Patients belonging to vulnerable groups (mentally ill, institutionalized, or under the age of 18)

This research was prepared in accordance with resolution 446 of the National Health Council, of 12/12/2012. All individuals involved in this study were instructed and signed the informed consent form (ICF) to participate. There was no conflict of interest. The project was approved by the Ethics in Research Committee of the Health Department of the Federal District under CAAE number 50965321.1.0000.5553 (opinion No. 5.196.206).

### Variables analyzed

The variables analyzed included age, sex, weight, height, BMI before bariatric surgery (kg/m^2^), BMI before reconstructive plastic surgery (kg/m^2^), total weight loss (kg), BMI variation (kg/m^2^), percentage of excess weight loss (%EWL), percentage of total weight loss (%TWL), time between bariatric and plastic surgeries (months), comorbidities before bariatric surgery, comorbidities before plastic surgery, number of medications used before and after bariatric surgery, types of surgical procedures in plastic surgery, weight of the abdominal flap removed after abdominoplasty, and postoperative complications in plastic surgery.

### Anthropometric variables

The percentage of excess weight loss (%EWL) was obtained from the formula: total weight loss after surgery/excess weight X 100. Excess weight was calculated by considering the weight at the beginning of the preoperative follow-up and subtracting from it the ideal weight, established by the BMI of 25 kg/m^2^. WE calculated the BMI variation (∆BMI) by the difference between the maximum pre-bariatric BMI and the BMI at the time of reconstructive plastic surgery. The %TWL was calculated by dividing the value of total weight loss (Kg) after the operation by the weight at the beginning of follow-up.

### Clinical variables and comorbidities

The diagnosis of systemic arterial hypertension, dyslipidemias, type 2 diabetes mellitus, and metabolic syndrome were based on the parameters contained in the respective guidelines of the Brazilian Society of Cardiology, currently described in the I Brazilian Guideline for the Diagnosis and Treatment of Metabolic Syndrome^7^.

The preoperative diagnosis of obstructive sleep apnea was made based on the apnea-hypopnea index (AHI), defined as the sum of apnea and hypopnea events per hour of sleep^8^. 

Arthropathy was defined as the patient who had undergone surgical treatment for joint pain or was regularly using anti-inflammatory drugs for the treatment of joint pain.

Anxiety disorder and depression were defined according to the criteria of the fifth edition of the Diagnostic and Statistical Manual of Mental Disorders (DSM-5)^9^.

### Reconstructive surgical procedures after gastroplasty

The reconstructive plastic surgeries performed in post-bariatric patients were abdominoplasty, mastoplasty, rhytidoplasty, brachioplasty, torsoplasty, and cruroplasty^10^.

Classic abdominoplasty included removal of excess skin and fat from the abdomen combined with wide detachment of the upper abdominal flap, correction of diastasis of the rectus abdominis muscles, and umbilical transposition. Anchor abdominoplasty, also called fleur-de-lis or “T” abdominoplasty, includes classic abdominoplasty associated with vertical resection at the midline level.

### Complication rate in plastic surgery in post-bariatric patients

Complications evaluated included hematomas, seromas, dehiscence, tissue necrosis, internal hernia, deep vein thromboembolism, and pulmonary embolism. Complications were divided into major and minor. Major complications were those requiring a new surgical procedure for hematoma drainage, seroma drainage, suturing of dehiscence areas, or rehospitalization for systemic antibiotic therapy.

### Statistical analysis

We performed statistical analysis with the Statistical Package for Social Sciences (SPSS) version 20.0 for Windows (SPSS Inc. Chicago, IL, USA). Continuous variables were described using mean and standard deviation, and categorical variables, relative frequencies. We assessed variables’ normality with the Kolmogorov-Smirnov test. The minimum significance accepted was 5% (p<0.05).

Comparisons between the groups were performed using the chi-squared test for dichotomous variables, the Student’s t-test for continuous variables with normal distribution, and the Mann-Whitney U test for continuous variables without normal distribution.

## RESULTS

The sample consisted of 316 post-bariatric patients who underwent plastic surgery procedures. All patients had previously undergone the same bariatric surgery technique, reduction gastroplasty with Roux-en-Y gastric bypass, 72.5% (229 patients) by laparoscopy and 27.5% (87 patients) by laparotomy. The mean age was 43.6 years ± 9.9 years (range 22-70), and females comprised 94.0% (297 patients) of the sample.

The mean maximum BMI before bariatric surgery was 45.47 ± 7.34 kg/m^2^. The mean maximum weight before bariatric surgery was 119.80 ± 21.65 kg. Patients undergoing bariatric surgery were more frequently obese grade III (80.7%), followed by grade II (19.3%).

Before the reconstructive plastic surgery, the mean BMI was 27.39 ± 3.91 kg/m^2^. The mean %EWL was 79.30% ± 12.91 and the mean %TWL, 39.55% ± 8.20. Patients at the time of plastic surgery were most frequently overweight (52.2%), followed by patients with normal BMI (25.6%) and residual obesity (22.2%).

Anthropometric variables are presented in Table 2. We observed that 31.6% (100/316) patients had a BMI variation greater than 20 and that 38.0% (120/316) had weight loss equal to or greater than 50kg.

**Table 2:** Demographic and anthropometric characteristics of the patients (N=316).

Variables	(Mean ± standard deviation)
Age, years	43.6 ± 9.9
Pre-gastroplasty max BMI	45.5 ± 7.3
Pre-plastic BMI	27.4 ± 3.9
Pre-gastroplasty weight, kg	119.8 ± 21.7
Pre-plastic weight, kg	72.2 ± 12.0
Post-gastroplasty weight loss, kg	47.4± 15.3
∆ BMI*	18.0 ± 5.7
%TWL	39.6±8.2
%EWL	79.3 ± 12.9

*BMI: Body Mass Index (kg/m2); ∆BMI: difference between current BMI and maximum BMI; %TWL: Percentage of total weight loss; %EWL: Percentage of excess weight loss.

Regarding the most prevalent comorbidities in the sample, systemic arterial hypertension (SAH) and metabolic syndrome were the most prevalent before bariatric surgery, present in 157 (49.7%) patients, followed by depression /anxiety (45.9%) and arthropathies (45.3%).

Most patients reported improvement or complete resolution of the various comorbidities after surgical treatment of obesity. However, some still had comorbidities at the time of reconstructive plastic surgery, with the presence of depression/anxiety in 98 (31%) individuals, followed by SAH and Arthropathy (Table 3).

**Table 3:** Distribution of patients according to the prevalence of comorbidities before and after gastroplasty, operated on at a Public Hospital in the Midwest, from 2011 to 2023, Brazil.

Associated comorbidities	Pre-gastroplasty n (%)	Post-gastroplasty n (%)	p-value	X2
Metabolic Syndrome	157 (49.7)	14(4.4)	<0.001	47.02
Hypertension	157 (49.7)	40 (12.7)	<0.001	53.02
Depression/Anxiety	145(45.9)	98(31.0)	<0.001	40.88
Arthropathy	143(45.3)	22 (7.0)	<0.001	46.01
Diabetes mellitus	137(43.4)	18(5.7)	<0.001	41.02
Sleep Apnea	78 (24.7)	3 (0.9)	0.015	27.04
Esophagitis	89 (34.1)	15(4.2)	<0.001	24.04
Dyslipidemia	95 (30.1)	8 (2.5)	0.001	25.04
Hepatic steatosis	122(38.6)	5(1.6)	0.050	21.05

There was a significant reduction in the number of pills used by patients before bariatric surgery compared with the ones used after bariatric surgery (4.95 ± 3.47 vs. 2.58 ± 1.91, p< 0.001, OR 4.95, 95%CI 4.57-5.33).

Two hundred and sixty-eight patients (84.8%) underwent only one surgical procedure per stage, while 48 (15.2%) underwent associated surgeries in the same surgical procedure.

Most patients underwent abdominoplasty (75.7%), the classic technique being the most applied, followed by the anchor technique. As for the other surgical procedures, mastoplasty was performed in 41.4% of patients, facial plastic surgery (rhytidoplasty) in 12.0%, arm plastic surgery (brachioplasty) in 12.0%, thigh plastic surgery (cruroplasty) in 5.7%, and dorsal plastic surgery (torsoplasty) in 5.1% of the patients (Table 4).

Incisional hernia repair was performed in 12 patients and umbilical hernia repair in 27, representing 12.3% of the patients who underwent abdominoplasty. Hernia repair was performed at the time of abdominoplasty.

The overall complication rate was 31.3%. The rate of major complications was 9.8% (31/316), while minor complications affected 21.5% (68/316) of the sample. As for the types of complications, wound dehiscence stood out in 12.0% (38/316) of the patients, seroma in 8.5% (27/316), wound infection in 5.1% (16/316), hematoma in 3.8% (12/316), intestinal obstruction due to internal hernia in 1.3% (4/316), and deep vein thrombosis in 0.6% (2/316) of the patients.

## DISCUSSION

Bariatric surgery is a valuable tool in weight loss and the improvement of several metabolic disorders, such as diabetes mellitus and dyslipidemia. The beneficial effects of bariatric surgery are maintained in the long term, as observed in individuals followed for more than ten years after the procedure, and translate into a reduction in cardiovascular events and mortality^4^ ^,^ ^11^. Thus, bariatric surgery is the treatment of choice for severely obese patients in whom behavioral and drug therapies has failed to control weight and comorbidities^11^.

However, after significant weight loss, complaints of tissue flaccidity and skin changes are frequent, mainly in the breasts, abdomen, back, arms, thighs, and face. This generalized dermatochalasis has an important psychosocial impact, as well as medical implications, such as intertrigo and functional limitations for ambulation, urination, and sexual activity^12^. 

Plastic surgery to repair body contouring helps to promote the social and psychological reintegration of these patients with already prolonged suffering. In addition, reconstructive plastic surgery aims to optimize the functional results obtained by bariatric surgery by removing excess skin^13^.

Reconstructive plastic surgery plays a key role in the long-term stabilization of the quality of life of patients with massive weight loss after bariatric surgery, maintaining its improvement in the long term^13^. The present study showed that most patients undergoing post-bariatric plastic surgery are women with a mean age of 43 years, a maximum BMI of 45.5kg/m^2^, a maximum mean weight of 119.8kg, and a mean weight loss of 47.4kg. Similar to national^14^ ^,^ ^15^ and other studies in Italy^16^, Austria^17^, France^18^, and Switzerland^19^. However, other studies have shown patients with a mean age higher than the one in our series, mainly in the United States^20^, Finland^5^, and Spain^21^, as well as a maximum BMI above 50kg/m², especially in the United States^20^ ^,^ ^22^. Regarding the high prevalence of post-bariatric procedures performed in women, we observed a statistically significant association between discomfort with excess skin after bariatric surgery and female sex, i.e., women are more bothered by excess skin after bariatric surgery than men^21^. In Brazilian public hospitals, women accounted for 95% of patients undergoing post-bariatric plastic surgery^23^. It is worth noting the significant increase in self-esteem and satisfaction with body image perception among post-bariatric women who undergo subsequent body contouring procedures^24^.

The mean BMI before plastic surgery was 27.4 kg/m^2^, similar to other Brazilian^12^ ^,^ ^15^ and international publications from Italy^16^, Austria^17^, France^18^, and Switzerland^19^. However, other studies conducted in the USA^20^ ^,^ ^25^, Finland^5^, and Turkey^26^ showed a mean BMI before plastic surgery above 30 kg/m². 

Overweight individuals represented 52.2% of our sample, while patients with normal BMI represented 25.6%, similar to the data of Orpheu et al.^14^, in which overweight individuals represented 56.1%, and patients with normal BMI, 16.32%.

Residual obesity is a persistent problem in patients after massive weight loss. Coon et al.^22^ pointed out that 45% of post-bariatric patients who seek plastic surgery for reconstructive operations ha residual obesity. We observed a prevalence of residual obesity of 22.2%, similar to that of another national study (27.6%)^14^.

There was a significant reduction in comorbidities after bariatric surgery, and at the time of reconstructive plastic surgery, only 12.7% of the patients had systemic arterial hypertension, and 5.7%, diabetes mellitus. In the USA, the prevalence of comorbidities before reconstructive plastic surgery in post-bariatric patients reaches 32.5% of arterial hypertension, 15% of diabetes mellitus, and 5% of sleep apnea syndrome^25^.

The significant improvement in the prevalence rates of comorbidities was directly reflected in the decreased use of medications after bariatric surgery. The mean number of pills taken before (4.95 ± 3.47) and after bariatric surgery (2.58 ± 1.91) was significantly different (p< 0.001, 95%CI 4.57-5.33).

Lopes et al.^27^ also found significant reductions in the number of pills used by patients after bariatric surgery. The mean number of medications used per patient decreased from 3.9 ± 1.67 medications before surgery to 1.64 ± 1.68 in the postoperative period. Therefore, a reduction of more than 50% in the number of drugs used per patient after surgery^27^.

The main post-bariatric reconstructive plastic surgery performed was abdominoplasty (80.4% of the patients), followed by mastoplasty, as corroborated by other studies in the United States^20^ ^,^ ^22^ and in Brazil^23^ ^,^ ^28^. 

In Brazil, there was a progressive growth in the number of plastic surgeries in post-bariatric patients, but below the demand and need of reference centers. In addition, the growth in the number of bariatric procedures was significantly higher than the number of post-bariatric plastic surgeries performed, according to data from the Department of Informatics of the Unified Health System (DATSUS)^28^.

According to DATSUS, between 2015 and 2020, the Midwest was the second last Brazilian region in number of post-bariatric plastic surgeries, after the Southeast, South, and Northeast regions, respectively. The Southeast region represents 53% of the total post-bariatric plastic surgeries performed in public hospitals in Brazil. One of the reasons is the high concentration of surgeons in this specialty, corresponding to 60% of these specialists registered in this region, according to a census conducted by the Brazilian Society of Plastic Surgery^23^.

Body contouring surgeries after massive weight loss are not without risks, as the procedures involve long skin incisions in patients who have large excess skin, as well as residual comorbidities and protein and vitamin deficiencies that interfere with healing.

The overall complication rate after plastic surgery in post-bariatric patients in the present study was 31%, similar to the studies by Kervilier et al.^19^ and Espinosa-de-los-Monteros et al.^20^, but below other studies with rates ranging from 35 to 50%^22^ ^,^ ^29^. Some risk factors have been associated with postoperative complications in post-bariatric patients undergoing reconstructive plastic surgery, such as high body mass index (BMI), sex, age, weight loss, smoking, amount of tissue removed, association of surgical procedures at the same stage, and presence of comorbidities^5^ ^,^ ^30^ ^,^ ^31^.

The rate of major complications in this study was acceptable, as we observed a small number of thromboembolic events, flap necroses, and reoperations. Some explanations can be pointed out, such as the low prevalence of associated procedures in the same operation in the sample. Studies that had higher complication rates had a higher percentage of surgical associations^22^. The association of surgeries leads to longer surgical time, greater blood loss, and greater need for blood transfusions, which favored an increase in the rate of postoperative complications in other studies^22^ ^,^ ^25^.

Another risk factor for postoperative complications is smoking, and some studies have reported smoking rates of up to 48% in patients undergoing abdominoplasty. We did not include smokers in the present study, considering smoking an exclusion criterion, since tobacco use significantly increases the risk of complications in the surgical wound^16^ ^,^ ^30^ ^,^ ^32^.

Another crucial factor that may have contributed to the lower complication rate was the low presence of comorbidities at the time of reconstructive plastic surgery. An American study involving 449 post-bariatric patients showed a complication rate of 41.8%. However, the prevalence of systemic arterial hypertension was 44.2%, and of diabetes mellitus, 22.3% among patients who underwent post-bariatric reconstructive plastic surgery^24^. 

The fact that most post-bariatric patients in our series had a BMI below 30kg/m^2^ is an anthropometric parameter that may have contributed significantly to the reduction of postoperative complications, as residual obesity can increase their rate, even having been adopted as an exclusion criterion (BMI greater than 30kg/m^2^) for body contouring surgery after bariatric surgery in plastic surgery services in some countries^5^.

On the other hand, a history of previous bariatric surgery would act as a protective factor, contributing to a decrease in postoperative complications, such as wound infection, dehiscence, and reoperations in obese patients undergoing procedures to remove excess skin from the abdomen compared with obese patients with no history of previous bariatric surgery^30^.

However, a meta-analysis study observed that the relative risk (RR) for the development of complications (especially of the surgical wound) after body contouring surgery between post-bariatric and non-post-bariatric patients with massive weight loss was 1.60 (95%CI 1.30-1.96, p<0.000001). This shows that there is a 60% increased risk of developing complications if the patient has lost weight surgically^33^. Thus, the main postoperative complication in the present study was surgical wound dehiscence, followed by seroma in abdominoplasties.

The advent of bariatric surgery has brought lasting and satisfactory results in the fight against obesity. The patient’s desire after massive weight loss is to perform surgical procedures to improve body contour. The surgeon’s careful and differentiated approach to each case, along with a multidisciplinary follow-up, will be fundamental for the appropriate management of these patients, aiming at the best reparative result and prevention of complications^34^.

Studies focusing on the clinical and surgical profile and complications of body contouring surgery after massive weight loss will help shift these reparative procedures into an integral part of the bariatric surgery process, as 92% of surgeons perceive that their post-bariatric patients face functional problems related to excess skin after massive weight loss. However, only 66% of surgeons routinely talk to their patients about these problems before bariatric surgery^35^.

Limitations of our study include its retrospective nature and patients coming from a single institution. The results may not be representative of Brazil’s Midwest, as all patients were operated on in a public referral hospital for the treatment of obesity. In addition, there may be a selection bias, as individuals who underwent associated procedures may have been the ones considered healthier and without comorbidities, while patients with comorbidities may have been selected for less complex procedures, with a lower risk of complications. Multicenter studies with a larger number of patients are essential to portray more accurately the clinical, anthropometric, and surgical profile of post-bariatric patients undergoing plastic surgery reconstructive procedures in Brazil.

Despite the significant weight loss of post-bariatric patients, they are unable to fully reverse their risk for postoperative complications^33^. At the time of body contouring surgery, factors such as nutritional status, current weight, BMI, and number of associated procedures need to be analyzed to reduce the risk of complications. In addition, weight stability after bariatric surgery brings better benefits in terms of local and systemic complications^34^.

## CONCLUSION

Roux-en-Y gastroplasty was an effective therapy in the resolution of comorbidities in obese patients. The most performed plastic surgery procedure in post-bariatric patients was abdominoplasty, followed by mastoplasty. The prevalence of residual comorbidities at the time of plastic surgery is low in these patients, especially hypertension. The postoperative complication rate was 31%, though most complications were considered minor, especially surgical wound dehiscence. 
